# Pb Single Atoms Enable Unprecedented Catalytic Behavior for the Combustion of Energetic Materials

**DOI:** 10.1002/advs.202002889

**Published:** 2021-01-04

**Authors:** Wengang Qu, Shiyao Niu, Da Sun, Hongxu Gao, Yishang Wu, Zhifeng Yuan, Xueli Chen, Ying Wang, Ting An, Gongming Wang, Fengqi Zhao

**Affiliations:** ^1^ Science and Technology on Combustion and Explosion Laboratory Xi'an Modern Chemistry Research Institute Xi'an Shaanxi 710065 China; ^2^ Hefei National Laboratory for Physical Science at the Microscale Department of Chemistry University of Science and Technology of China Hefei Anhui 230026 P. R. China

**Keywords:** energetic materials, orbital level engineering, PDA‐Pb, single atoms, thermal decomposition

## Abstract

Manipulating the thermal decomposition behavior of energetic materials is the key to further pushing the combustion performance of solid rocket propellants. Herein, atomically dispersed Pb single atoms on polydopamine (PDA‐Pb) are demonstrated, which display unprecedented catalytic activity toward the thermal decomposition of cyclotrimethylenetrinitramine (RDX). Impressively, RDX‐based propellants with the addition of PDA‐Pb catalyst exhibit substantially enhanced burning rates (14.98 mm s^−1^ at 2 MPa), which is 4.8 times faster than that without PDA‐Pb and represents the best catalytic performance among Pb‐based catalysts. Moreover, it also possesses low‐pressure exponents in broad pressure ranges, which can enable more stable and safer combustion in solid rocket engines. Theoretical calculation unravels the efficient catalytic activity is stemmed from the enhanced interfacial electronic coupling between RDX and PDA‐Pb via orbital level engineering. More importantly, PDA‐Pb also presents similar catalytic behavior toward the decomposition of nitrocellulose, suggesting its broad catalytic generality. This work can open up new opportunities in the field of energetic compound combustion by exploring Pb‐based single atom catalysts and beyond.

## Graphical abstract

Atomically dispersed Pb single atoms on polydopamine (PDA‐Pb) display excellent catalytic activity towards the thermal decomposition of energetic materials. The addition of PDA‐Pb can substantially decrease the thermal decomposition temperature and consequently boost the burning rate of the propellants. Theoretical calculations reveal the efficient catalytic activity is stemmed from the enhanced interfacial electronic coupling via orbital level engineering.

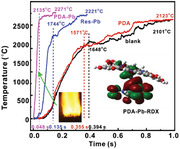

## 1 Introduction

Energetic compounds such as cyclotrimethylenetrinitramine (RDX), cyclotetramethylene tetranitramine, hexanitrohexaazaisowurtzitane, and nitrocellulose (NC), have been widely used in explosives, pyrotechnics, gun propellant, and solid rocket propellants as the main energetic fillers.^[^ ^1^ ^]^The thermal decomposition behavior of the energetic compounds plays vital effects on the combustion efficiencies of the propellants and further determines the performance of the rocket propulsion systems.^[^ ^2^ ^]^ In this regard, improving the thermal decomposition efficiencies of these energetic materials and understanding the combustion behavior is the key to pushing the performance of the solid rocket propulsion systems. Currently, the most commonly used strategy to enhance the thermal decomposition efficiency of the energetic compounds is adding combustion catalysts, such as metal, metal oxides, or metal salts.^[^ ^3^ ^]^ Among these catalysts, Pb‐based catalysts have been extensively used in the solid propellant, with a history of more than 70 years.^[^ ^4^ ^]^ For example, it has been reported that CuO/PbO composite catalysts exhibit an impressive burning rate of 8.33 mm s^−1^ at 2 MPa in the catalytic combustion of RDX‐based propellant.^[^ ^5^ ^]^ Given that the catalytic process typically takes place on the interface between the catalysts and energetic compounds, modulating the interfacial electronic coupling to effectively activate the energetic compounds is highly critical.^[^ ^6^ ^]^

With the development of nanotechnology, nanocatalysts for catalytic combustion of energetic materials recently raise great interest, due to their large surface area and adjustable surface electronic properties.^[^ ^7^ ^]^ However, the practical application of nanocatalysts in solid propellants is severely impeded by the easy agglomeration and the poor dispersibility, which inevitably leads to the inhomogeneous dispersion of catalysts in the solid propellant and consequently result in uncontrollable combustion and instable propulsion systems.^[^ ^8^ ^]^ Although loading the nanocatalysts on carbon supports such as graphene oxides and carbon nanotubes can alleviate this issue to some extent, the overall combustion performance is still far from being satisfactory.^[^ ^5^, ^9^ ^]^ Moreover, the weight ratio of the added catalyst is also relatively high in the propellants, leading to the decreased energy density of the whole propulsion systems. To this end, it is highly desirable to develop efficient combustion catalysts with minimum catalyst loading ratio and maximum interfacial electronic activation. Meanwhile, unraveling the catalytic essence is also equally important, which could provide valuable insights for the rational design of catalysts in the future.^[^ ^10^ ^]^

Herein, we first demonstrate atomically dispersed Pb single atoms anchored on the polydopamine (PDA‐Pb) exhibit exceptional catalytic activity for the thermal decomposition of the RDX by coating PDA‐Pb on its surface directly. The Pb single‐atom catalysts can not only minimize the utilization of metal in the combustion catalysts, but also the PDA with the unparalleled adhesive property enables an intimate contact between the catalytic sites and the energetic materials.^[^ ^11^ ^]^ Impressively, with the addition of the PDA‐Pb catalysts, the RDX‐based propellants exhibit unprecedented burning rates (14.98 mm s^−1^ at 2 MPa), which is the best burning rate among the ever‐reported combustion catalysts.^[^ ^12^ ^]^ More importantly, the burning rate is also insensitive to the pressure variations with an impressive pressure exponent lower than 0.2 from 2 to 20 MPa, which is highly favorable and important for developing stable and efficient solid rocket propulsion systems. Moreover, density functional theory (DFT) calculation reveals single Pb atoms‐based catalysts with superior energy levels can well activate the RDX molecules for catalytic decomposition, by enhancing the interfacial electronic coupling between RDX and PDA‐Pb. This work represents the first example to use single‐atom catalysts for boosting the combustion efficiencies of the energetic compounds, which could offer new opportunities to explore single‐atom catalysts in combustion chemistry of the solid rocket propellant.

## 2 Results

**Figure 1:**
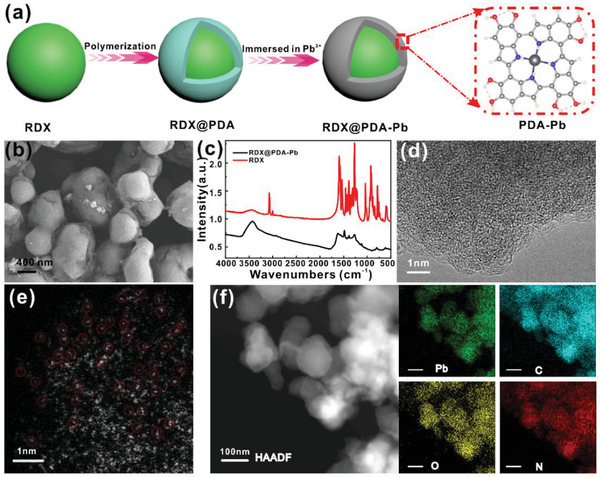
The synthesis and characterization of RDX@PDA‐Pb. a) The schematic illustration of the synthesis of PDA‐Pb coating layer on the RDX. b) SEM image of the RDX@PDA‐Pb. c) The FTIR spectra of the RDX@PDA‐Pb and RDX. d) High‐resolution TEM image of the PDA‐Pb polymer layer. e) High‐resolution HAADF‐STEM image of the PDA‐Pb to reveal the distribution of the single Pb atoms. f) The HAADF‐STEM image and the corresponding elemental mapping images of Pb (green), C (blue), O (yellow) and N (red), respectively.

The synthesis of PDA‐Pb coated RDX (denoted as RDX@PDA‐Pb) is achieved through a facile two‐step incubation process, as depicted in **Figure** **1**a. First, the PDA layer is coated on the surface of RDX particles via a simple in situ polymerization of dopamine.^[^ ^13^ ^]^ Then, Pb ions are anchored on the PDA‐coated RDX through the chelating interactions between the amine groups of the PDA and the Pb ions. Scanning electron microscopy (SEM) is employed to characterize the morphology of the as‐prepared samples, as shown in Figure 1b. Clearly, the RDX particles are uniformly encapsulated within a thin polymer shell, revealing an intimate contact between the RDX and the PDA‐Pb coating shell. Figure 1c displays the Fourier‐transform infrared spectra (FTIR) of the RDX@PDA‐Pb and the RDX. Apparently, with the coating layer, the FTIR spectrum of the RDX@PDA‐Pb exhibits the characteristic features of the PDA‐Pb, indicating the successful polymerization of dopamine on the surface of RDX particles.^[^ ^14^ ^]^ Furthermore, high‐resolution transmission electron microscopy (HRTEM) and aberration‐corrected high‐angle annular dark‐field scanning transmission electron microscopy (HAADF‐STEM) are used to study the surface coating structures of PDA‐Pb. Impressively, the coated polymer layer exhibits an amorphous feature (Figure 1d) and the Pb are atomically dispersed on the PDA coating (Figure 1e). Other HAADF‐STEM images with low magnification are shown in Figure S1, Supporting Information. The uniform contrast in the low‐magnification image suggests that there is no obvious other phase incorporated in the PDA‐Pb. Inductively coupled plasma atomic emission spectroscopy analysis reveals the Pb content in the PDA‐Pb is around 1.2 wt%. Meanwhile, the energy‐dispersive X‐ray spectroscopy mapping in Figure 1f indicates the uniform distribution of Pb, C, O, and N, also suggesting the homogeneous dispersion of Pb single atoms on the polymer coating layer. Taken together, all these results clearly demonstrate atomically dispersed Pb atoms are homogeneously anchored on the PDA polymer layer, which enables intimate contact with the inner energetic RDX particles.

**Figure 2:**
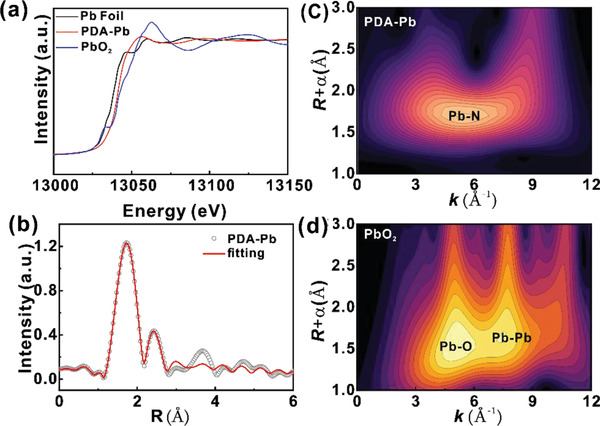
XAFS analysis of the PDA‐Pb catalysts. a) The XANES Pb L_3_‐edge spectra and b) the Fourier transform (FT) EXAFS spectra of PDA‐Pb. The contour plots of the WT spectra of c) PDA‐Pb, d) PbO_2_, respectively.

In light of the amorphous feature of the PDA‐Pb, we further perform synchrotron‐based X‐ray absorption fine structure spectroscopy (XAFS) and X‐ray photoelectron spectroscopy (XPS) to probe the chemical and coordination states of Pb single atoms in the PDA‐Pb. **Figure** **2**a displays the X‐ray absorption near‐edge structure (XANES) Pb L_3_‐edge spectra of the Pb foil, PDA‐Pb, and PbO_2_, in which Pb foil and PbO_2_ are used as reference samples. Clearly, the white line intensity of PDA‐Pb is higher than the Pb foil, suggesting that the chemical states of the Pb in PDA‐Pb are oxidized not metallic.^[^ ^15^ ^]^ Meanwhile, the white line intensity of PDA‐Pb is lower than that of PbO_2_, indicating the oxidation state of the Pb in PDA‐Pb is lower than that in PbO_2_, due to the stronger electronegativity of oxygen than the nitrogen in amine groups of the PDA.^[^ ^16^ ^]^ In addition, the high‐resolution XPS Pb 4f spectrum of PDA‐Pb can be deconvoluted into two peaks located at 138.2 and 143.1 eV, which can be assigned to the Pb 4f_7/2_ and Pb 4f_5/2_ of the Pb—N bonds, respectively (Figure S2, Supporting Information). Figure 2b shows the R space of the extended X‐ray absorption fine structure (EXAFS) for the PDA‐Pb, which exhibits prominent peaks at 1.8 and 2.2 Å originated from the Pb–N shell. Since the XAFS provides the average local structural information, it can be concluded that the Pb in PDA‐Pb is atomically dispersed by combining with the HAADF‐STEM data in Figure 1e. Meanwhile, we also conduct the EXAFS fitting in the range of 1.0 to 3 Å using Pb foil as the reference, and the EXAFS fitting of the reference Pb foil is provided in Figure S3, Supporting Information. The details of the fitting parameters are presented in Table S1 and Figure S4, Supporting Information. The PDA‐Pb possesses prominent peaks located at 2.46 and 2.70 Å from the Pb–N shell with a coordination number of 2 and 2, respectively (Table S1, Supporting Information).^[^ ^17^ ^]^ Also, the Pb—N coordination shown in Table S1, Supporting Information, implies the Pb is atomically dispersed in the PDA matrix. Furthermore, the wavelet transform (WT) of Pb L_3_‐edge EXAFS oscillation is a powerful way to precisely illustrate the coordination structures, by providing not only radial distance resolution but also k‐space resolution.^[^ ^18^ ^]^ Figure 2c,d displays the WT contour spectra of PDA‐Pb and the control sample of PbO_2_. In comparison with the Pb‐O vector (*k* = 5.1 Å^−1^) and Pb‐Pb vector (*k* = 7.9 Å^−1^) in PbO_2_, the intensity maxima only centered at *k* = 5.5 Å^−1^ are attributed to the Pb—N bonds in the PDA‐Pb, suggesting that the Pb species is atomically dispersed in PDA with different coordination states from the PbO_2_. Taken together, fine structure analysis clearly indicates the successful preparation of atomically dispersed Pb atoms on the PDA matrix.

**Figure 3:**
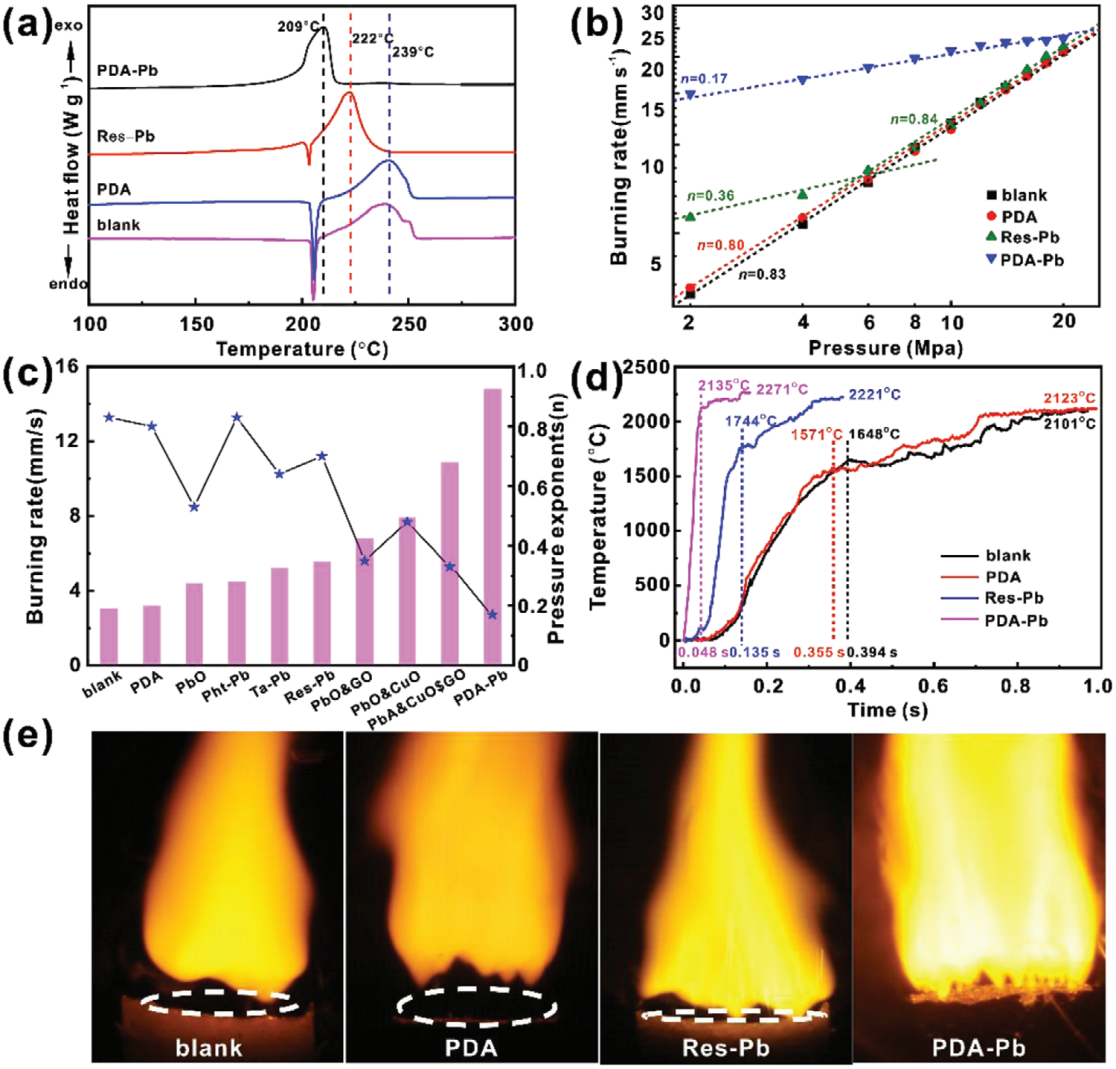
The catalytic effects of Pb‐PDA for the thermal decomposition of the RDX. a) The DSC profiles of RDX, RDX@PDA‐Pb, RDX@PDA, and RDX/Res‐Pb. b) The dependence of the burning rate on the pressure with the various catalyst additives. c) The comparison of the burning rate of RDX@PDA‐Pb with the ever‐reported combustion additives. d) The combustion temperature profiles and e) the combustion flames of the blank propellants and the propellants with the additives of PDA, Res‐Pb, and PDA‐Pb, respectively.

To evaluate the catalytic effects of PDA‐Pb on the thermal decomposition properties of the energetic RDX molecules, differential scanning calorimetry (DSC) analysis is carried out with a heating rate of 10 °C min^−1^ under the flow of nitrogen. The organic salt of lead resorcinol acid (denoted as Res‐Pb), a commonly used catalyst additive for energetic compound combustion, is taken for comparison, while PDA without loading Pb single atoms is used as a control sample. The additives of Res‐Pb and RDX are homogeneously mixed with a weight ratio of 1:10 by grinding the mixed powders with a mortar and pestle. **Figure** **3**a displays the representative DSC curves of pure RDX, RDX@PDA‐Pb, RDX@PDA, and RDX/Res‐Pb. Impressively, a significant difference in the decomposition temperature is observed for the RDX with different additives. The pure RDX exhibits two decomposition peaks, which represent the melting peak from the solid to liquid phase at 204 °C and the decomposition peak at 239 °C. Only with the addition of PDA, the DSC profiles of RDX can be well maintained, suggesting PDA has no catalytic effect on the thermal decomposition of RDX. In comparison, with the addition of Res‐Pb, the decomposition temperature is reduced from 239 °C for pure RDX to 222 °C for RDX/Res‐Pb. It suggests Res‐Pb has an excellent catalytic property for the decomposition of RDX molecule, which is the reason why it has been extensively used as the combustion additives in the propulsion systems. Excitingly, in the presence of PDA‐Pb coating, the melting peak disappears and the temperature of the decomposition peak is substantially reduced to 209 °C. The absence of the melting peak indicates PDA‐Pb possesses unprecedented catalytic activity towards the activation of RDX molecule, which thus enables fast catalytic decomposition reaction. More importantly, the 209 °C is also the ever‐reported lowest decomposition peak temperature for RDX under the same operating condition.^[^ ^12^ ^]^ Interestingly, we also find that the PDA‐Pb also displays similar catalytic behavior toward the thermal decomposition of NC (Figure S5, Supporting Information), suggesting the broad generality and great promise of single‐atom catalysts for the thermal decomposition of the energetic compounds.

To demonstrate whether the RDX@PDA‐Pb could be practically applied in the solid propellant, we choose a mature solid propellant formula as a research model with RDX@PDA‐Pb as the main energetic filler, NC as the binder, and nitroglycerine as the plasticizer. Meanwhile, blank RDX without catalysts, RDX @PDA, and the lead salt of res‐Pb are used as control samples. The propellant samples are prepared by using the industrial solvent‐free extruding method. Figure 3b shows the dependence of the burning rate on the pressure and the detailed data are summarized in Tables S2 and S3, Supporting Information. The burning rate (*r*) a typical parameter to evaluate the energy releasing rate of propellant, is also described as the regression rate of the burning surface of the solid propellant, while the pressure exponent (*n*) defines the dependence of the burning rate on pressure (*p*), which can be well expressed by Vieille's law.^[^ ^19^ ^]^ When only using RDX as the energetic filler, the burning rate at the initial pressure of 2 MPa is 3.09 mm s^−1^ and then increases rapidly as the pressure further elevates. The pressure exponents on each pressure point are consistently over 0.7 and the average value of *n* on the whole pressure range is ≈0.83. With the PDA as the additive, the burning rate does not show obvious enhancement, suggesting PDA does not have catalytic effects on the combustion of RDX‐based propellant. However, when incorporated with the Res‐Pb, the burning rate can be substantially enhanced, achieving a burning rate of 5.65 mm s^−1^ at 2 MPa. The *n* in the pressure range of 2–6 MPa is reduced to 0.36 with the Res‐Pb. When the pressure is larger than 10 MPa, the burning rate–pressure curve almost coincides with the blank sample. As a comparison, the burning rate of the sample using RDX@PDA‐Pb at 2MPa is 14.98 mm s^−1^, which is 4.8 times and 2.65 times faster than those of the blank RDX and the RDX/Res‐Pb. To the best of our knowledge, the obtained burning rate represents the highest performance among the ever‐reported additives for energetic compounds (Figure 3c).^[^ ^12^ ^]^ Typically, high burning rates are often accompanied by a high value of the pressure exponent, which could potentially lead to catastrophic consequences.^[^ ^20^ ^]^ Unexpected, the pressure exponent of RDX@PDA‐Pb sample on each pressure point is consistently lower than 0.3 and the average value of *n* in the pressure range from 2 to 20 MPa is only 0.17, which is significantly important for the development of more stable and safe rocket engines. Such a wide combustion plateau indicates that the combustion process in the whole pressure range is under reaction control rather than the diffusion control,^[^ ^21^ ^]^ which is intrinsically attributed to the high catalytic activity of the PDA‐Pb and thus enables stable combustion in such broad pressure range.

To further investigate the combustion performance of the prepared propellants, the combustion temperature profiles and the combustion flames of the propellants with different additives are recorded at 2 MPa, as shown in Figure 3d,e. For the blank sample, the temperature first rises quickly to 1648 °C (temperature of dark zone: *T*_d_) within 0.394 s in the solid‐phase zone and fizz zone, and then slowly rises to the final maximum temperature of 2101 °C (*T*_max_), which is accompanied by a “dark zone” in the combustion flame (highlighted in Figure 3e), a typical region of the propellant combustion with slow exothermic reactions.^[^ ^22^ ^]^ Similarly, the PDA sample exhibits a very close temperature profile, indicating that PDA has no catalytic effects on the combustion of the propellant. For the Res‐Pb sample, the dark zone is distinctly shortened and the temperature rises more rapidly, which can reach 1744 °C (*T*_d_) in 0.135 s, mainly due to the accelerated thermal decomposition induced by the Res‐Pb. Impressively, the sample with PDA‐Pb displays completely different combustion behavior. The dark zone almost disappears and the combustion reaction occurs directly on the burning surface. Besides, the temperature rises directly to 2135 °C in only 0.048 s and the *T*_max_ is also greatly improved to 2271 °C, meaning the PDA‐Pb catalyst enables much faster and stronger energy release. Together, the highest catalytic combustion and lowest pressure exponent endow RDX@PDA‐Pb with great promise for the practical solid propellant system.

**Figure 4:**
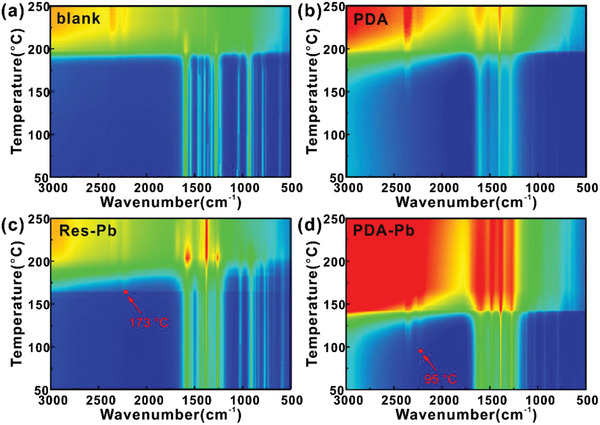
In situ FTIR studies on the decomposition of RDX. The FTIR spectra of the thermal decomposition of a) blank RDX and RDX with the addition of b) PDA, c) Res‐Pb, and d) PDA‐Pb, at the varying temperatures.

We further conduct in situ FTIR spectroscopy measurements with a heating rate of 10 °C min^−1^ to investigate the catalytic decomposition of RDX on PDA, Res‐Pb, and PDA‐Pb, as shown in **Figure** **4**. A sudden rise in the spectral background is due to the incident light scattering caused by the gas evolution during the RDX decomposition. Accordingly, the initial decomposition temperatures for both pure RDX and RDX@PDA start at 190 °C (Figure 4a,b), while the decomposition reaction of the RDX@PDA‐Pb and RDX/Res‐Pb are initiated at 140 and 180 °C (Figure 4c,d), respectively. The decreased initial reaction temperature means that the RDX molecule is activated by PDA‐Pb and Res‐Pb and the corresponding decomposition kinetics are boosted. The in situ FTIR spectra of RDX@PDA‐Pb and RDX/Res‐Pb exhibit the characteristic peak located at 2200–2250 cm^−1^ at 95 and 173^ ^°C, which correspond to the stretching vibration of N_2_O (Figure 4c,d). Meanwhile, the absorption bands at 1271, 1303, 2204, and 2238 cm^−1^ of the FT‐IR spectra of the gaseous products is attributed to the generation of N_2_O, while the bands at 1599 and 1630 cm^−1^ are stemmed from NO_2_ (Figures S6–S9, Supporting Information). According to previous reports, the generation of N_2_O and NO_2_ should originate from the bond breakage of the N–NO_2_ in RDX.^[^ ^23^ ^]^ Obviously, the generation of N_2_O and NO_2_ can be well detected during the thermal decomposition of RDX/Res‐Pb and RDX@PDA‐Pb at a lower temperature range than those of pure RDX and RDX@PDA, which is due to the more effectiveness in the RDX activation with the Pb‐based catalysts. The addition of Pb‐based compounds could trigger the breakage of the N–NO_2_, which further leads to fast decomposition reactions of RDX molecules.

**Figure 5:**
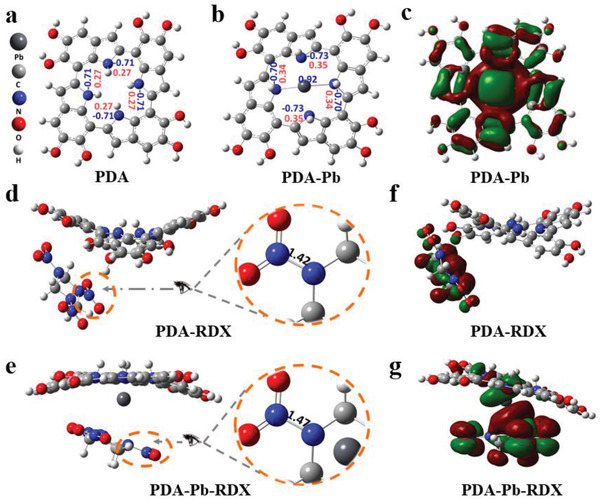
Theoretical analysis of the modulation essence of Pb‐PDA on the decomposition of RDX molecule. The top‐view structure of a) PDA and b) PDA‐Pb with labeled mulliken charge distributions. c) The HOMO of the PDA‐Pb. d) The bond lengths of N—NO_2_ in the PDA‐RDX and e) PDA‐Pb‐RDX. f) The HOMO of PDA‐RDX and g) PDA‐Pb‐RDX.

To understand the fundamental modulation essence of PDA‐Pb on the thermal decomposition of RDX at the atomic level, we conduct DFT calculations. **Figure** **5**a,b shows the top‐view structures of the PDA and the PDA‐Pb, in which the Pb ion is anchored by four ‐NH groups of the PDA molecules. Meanwhile, the mulliken charge distributions of N and H are also highlighted in the structure diagram. With the coordination between Pb ion and PDA molecules, the overall charge density of the four NH is reduced, while the charge density around on Pb^2+^ is enhanced, indicating that obvious charge transfer occurs between Pb^2+^ and PDA molecules. Figure 5c illustrates the highest occupied molecular orbital (HOMO) of PDA‐Pb. Conspicuous orbital overlap takes place between Pb^2+^ and PDA, which means Pb ions can be chemically anchored by the PDA molecules and it is also consistent with the results of charge distribution analysis. In addition, the large binding energy of −56.3 eV also indicates that Pb ion has been well riveted to PDA with stable structural configuration. Since in situ FTIR studies reveal that the N—NO_2_ bond in RDX is first broken during the initial decomposition, we focus on the catalytic effects of PDA‐Pb on the bonds of N–NO_2_ in the RDX. Figure 5d,e shows the changes of bond length of N–NO_2_ in RDX with and without Pb single atoms, and the charge distribution of NO_2_ group and Pb ion are presented in Figure S10, Supporting Information. The bond length of N–NO_2_ (Figure S11, Supporting Information) and charge distribution of NO_2_ (Figure S12, Supporting Information) in the pure RDX are presented for comparison. Impressively, the bond length of N–NO_2_ in RDX molecule is elongated obviously on the PDA‐Pb, while the bond length is almost unchanged on the PDA. Meanwhile, more charge is outflowed from the NO_2_ group in RDX to PDA‐Pb, indicating PDA‐Pb plays a vital role in catalytic activation of the N—NO_2_ bond in RDX. Furthermore, Figure 5f,g reveals there is more orbital overlap in the HOMO of PDA‐Pb‐RDX than that in PDA‐RDX, indicating stronger active charge transfer between RDX and PDA‐Pb, which is beneficial to the activation of the N—NO_2_ bond.

Frontier molecular orbital theory is finally conducted to analyze the energy level distribution of each molecular orbital and further analyze the essence of catalysis. Figures S13 and S14, Supporting Information, illustrate the relative positions of the lowest unoccupied molecular orbital (LUMO) and HOMO of PDA, RDX, PDA‐RDX, RDX‐Pb, and PDA‐Pb‐RDX. Basically, to activate the RDX molecule, it requires the HOMO of the PDA or PDA‐Pb to attack the LUMO of RDX to generate charge interaction. It can be seen from the orbital energy levels of PDA‐RDX (Figure S13, Supporting Information), in which the LUMO of PDA‐RDX is basically flat with the LUMO of RDX, and the HOMO of PDA‐RDX is basically flat with the HOMO of PDA. Therefore, the orbital of PDA‐RDX is just the simple superposition of PDA and RDX, indicating that there is no substantial interaction between them. As shown in Figure S14, Supporting Information, with the addition of Pb ion, the orbital level of PDA‐Pb drops significantly compared to that of PDA, leading to a close position between the LUMO of PDA‐Pb and the HOMO of RDX, which thus makes it easier for the HOMO electrons of RDX to attack the LUMO orbital of PDA‐Pb and form orbital coupling between them. This is also consistent with the electron flow from RDX to the Pb, as shown in Figure S10, Supporting Information. Therefore, the PDA‐Pb essentially reduces the energy level positions of HOMO and LUMO in PDA, and finally promotes the activation of the N—NO_2_ bond of the RDX molecules by enhancing the interfacial electronic coupling.

## 3 Conclusion

In summary, we have demonstrated the atomically dispersed Pd single atoms anchored on the PDA matrix own unprecedented catalytic activity for the activation and thermal decomposition of the RDX molecules. The RDX@PDA‐Pb‐based solid propellant displays an ultrafast burning rate, which is 4.8 times higher than the RDX counterpart and also sets a new record of burning rate among the ever‐reported Pb‐based catalysts. More impressively, the solid propellant with the RDX@PDA‐Pb also exhibits low‐pressure exponents under wide pressure ranges, which holds great promise for developing a stable and safe propulsion system. Theoretical analysis reveals the unexpected high activity of PDA‐Pb is originated from the enhanced electronic coupling between RDX molecules and PDA‐Pb via orbital level engineering. This work demonstrates the first example to explore a single‐atom catalyst for boosting the energetic compound combustion, which could raise new opportunities to develop efficient and stable solid rocket propulsion systems.

## 4 Experimental Section

Experimental Section is available in the Supporting Information.

## Conflict of Interest

The authors declare no conflict of interest.

## Supporting information

Supporting Information
