# Supplementary material for: Pb Single Atoms Enable Unprecedented Catalytic Behavior for the Combustion of Energetic Materials
Source: Adv Sci (Weinh). 2021 Jan 4;8(5):2002889. doi: 10.1002/advs.202002889 (PMC7927613; doi:10.1002/advs.202002889)
Supplement: Supplementary file 1 — Supporting Information [file ADVS-8-2002889-s001.pdf]

WILEY-VCH

© 2020 Wiley-VCH GmbH

ADVANCED SCIENCE
Open Access

Supporting Information

for *Adv. Sci.,* DOI: 10.1002/advs.202002889

Pb Single Atoms Enable Unprecedented Catalytic Behavior for the Combustion of Energetic Materials

Wengang Qu,[1] Shiyao Niu,[1,2] Da Sun,[2] Hongxu Gao,[1] Yishang Wu,[2] Zhifeng Yuan,[1] Xueli Chen,[1] Ying Wang,[1] Ting An,[1] Gongming Wang,*[2] Fengqi Zhao*[1]*

((Supporting Information can be included here using this template))

© 2020 Wiley-VCH GmbH

Supporting Information

Pb single atoms enable unprecedented catalytic behavior for the combustion of energetic materials

Wengang Qu,[1] Shiyao Niu,[1,2] Da Sun,[2] Hongxu Gao,[1] Yishang Wu,[2] Zhifeng Yuan,[1] Xueli Chen,[1] Ying Wang,[1] Ting An,[1] Gongming Wang,*[2] Fengqi Zhao*[1]*

Experimental section

Materials: The dopamine hydrochloride (DOPA-HCL, 98%) and Tris(hydroxymethyl)aminomethane (Tris, 99%) were purchased from Sigma-Aldrich Co. The cyclotrimethylenetrinitramine was supplied by Xi'an Modern Chemistry Research Institute. The other reagents were obtained from Aladdin (Shanghai, China). All reagents were used without further purification.

Synthesis of RDX@PDA-Pb and RDX@PDA

The preparation of RDX@PDA: 20 g of RDX was added into 250 mL of a dopamine-Tris solution (2.0 $g \cdot L^{-1}$, pH=8.5, 10 mM Tris-HCl buffer) under stirring for 6 h at room temperature. The products were centrifuged, washed with deionized water for several times to get rid of the unreacted dopamine, and finally dried in a vacuum oven at 60 °C to obtain RDX@PDA.

The preparation of RDX@PDA-Pb: 20 g of RDX was added into 250 mL of a dopamine-Tris solution (2.0 $g \cdot L^{-1}$, pH=8.5, 10 mM Tris-HCl buffer) under stirring for 6 h at room temperature. The products were centrifuged, washed with deionized water for several times to get rid of the unreacted dopamine, and re-dispersed into 250ml deionized water solution, followed by adding 8.7 ml $Pb(NO_3)_2$ aqueous solution ($1mg \cdot mL^{-1}$). The solution was stirred

for 8 h, and the products were centrifuged, washed by deionized water for several times, and finally dried in a vacuum oven at 60 °C to obtain RDX@PDA-Pb.

Material characterization:

The crystalline phases of the prepared samples were recorded by X-ray diffraction (XRD, Philips, X'pert X-ray diffractometer with Cu K□). The products morphologies were analyzed by scanning electron microscopy (SEM, JEOL-JSM-6700F), transmission electron microscopy (TEM, JEM-2011) and high-resolution transmission electron microscopy (HRTEM, Talos F200X). The percentages and the distribution of the individual elements were measured by energy dispersive X-ray spectroscopy (EDX, JEM-ARM 200F) elemental mapping images and inductively coupled plasma atomic emission spectroscopy (ICP-AES, Optima 7300 DV). The X-ray photoelectron spectroscopy (XPS) was performed at photoemission end-station (BL10B) in the National Synchrotron Radiation Laboratory (NSRL), Hefei. The binding energy of XPS was calibrated based on the C1s peak at 284.6 eV. XAFS spectra of Pb L_3-edge (E_0=13035 eV) were conducted at the beamline (BL14W1) of Shanghai National Synchrotron Radiation Facility (SSRF, China). The XAFS data on PDA-Pb recorded under transmission mode operated at a double-crystal Si(111) monochromator. The acquired EXAFS data were normalized according to the standard procedures the ATHENA module implemented in the IFEFFIT software packages. The k^3-weighted $\chi(k)$ data of Pb L_3-edge were Fourier transformed to real (R) space to analyze the EXAFS contributions from the different coordination shells. The quantitative structural parameters of the studied sample were obtained by using ARTEMIS code. The energy of Pb was calibrated according to the absorption edge of pure Pt foil of L_2-edge (13273 eV). The passive electron factors, S_0^2, were determined by fitting the experimental Pb foil data and fixing the Pb-Pb coordination number (CN) to be 8 and 6, and then fixed for further analysis of the measured samples. The parameters describing the local structure environment such as CN, Debye-Waller (DW) factor and bond distance (R) around the absorbed Pb atoms could change during the fit process.

Differential scanning calorimetry (DSC) examinations were used to characterize the thermal behaviors, performed on a Netzsch HP 204 thermal analyzer under 50 ml·min^{-1} Ar atmosphere with attempted heating rating of 10 °C·min^{-1}. About 1.0 mg of sample was loaded into an Al crucible and heated from room temperature to 400 ℃. The vibrational characteristics of chemical bonds were determined using a Bruker Tensor 27 Fourier Transform Infrared (FTIR) spectrometer. The spectra of the samples (as KBr pellets with a KBr to sample mass ratio of approximately 30:1) were acquired in the range of 4000–400 cm^{-1} in the transmission mode with a resolution of 4 cm^{-1}.

For all the combustion property evaluations, a nickel–chromium alloy wire (~ 0.15 mm) was utilized for ignition in the experiments. The flame profiles were recorded by a camera using the single frame amplification photography method. The combustion wave distributions of propellant samples were obtained using the Π type double tungsten-rhenium thermocouple (Φ=25 μm), which was embedded in the propellant sample (diameter = 7 mm, length = 120 mm). During the combustion process, the burning surface moves gradually to the thermocouple, and finally, the thermocouple gets into the flame zone. Thus, the whole combustion process was recorded and the combustion wave structure from the condensed phase to gas phase was obtained.

Strand burning rates of the propellants were determined at pressures of 2–20 MPa by utilizing the acoustic emission technique. This method involves the combustion of propellant samples with dimensions of 150 mm × 5 mm × 5 mm in a stellar bomb filled with pressurized nitrogen and water.

Computation method:

The DFT simulations are performed using quantum chemical calculation software Gaussian 16. Geometries were fully optimized to lowest energies without symmetry restrictions. The non-restrictive Ubp86 mixed density functional method was applied for all atoms. LANL2DZ was used as the pseudopotential basis set for the Pb^{2+}, and the orbital splitting basis set 6-

311++g** was used for the other elements. Meantime, the corresponding vibration frequencies of all the steady-state structures were subjected to verify the nature of the stationary points. Equilibrium geometries are characterized by the absence of imaginary frequencies. Supposing that A and B are bound together, the binding energy E(bind) between them is calculated by $\mathrm{E(bind)} = E(\mathrm{A/B}) - \mathrm{E(A)} - \mathrm{E(B)}$, where E(A) and E(B) are the total energies of A and B, and E(A/B) is the total energy of the combined structure. And a more negative $E(\mathrm{A/B})$ indicates a more stable adsorption.

Supporting Figures:

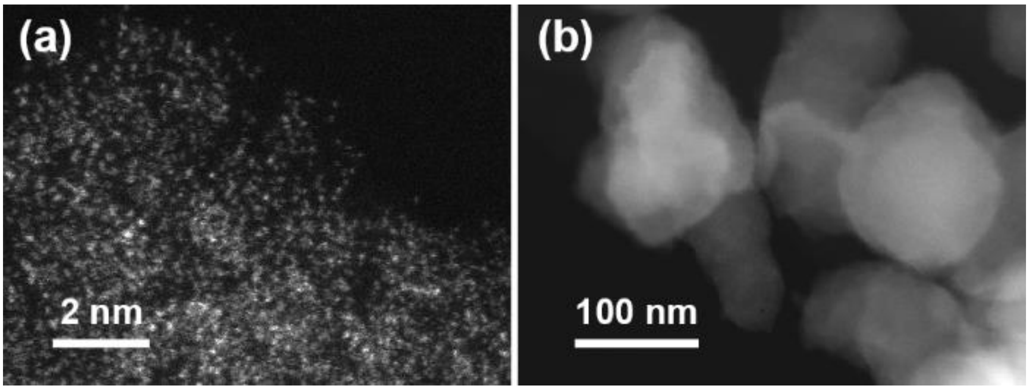

Figure S1. The (a) high and (b) low magnification HAADF-STEM images of the PDA-Pb.

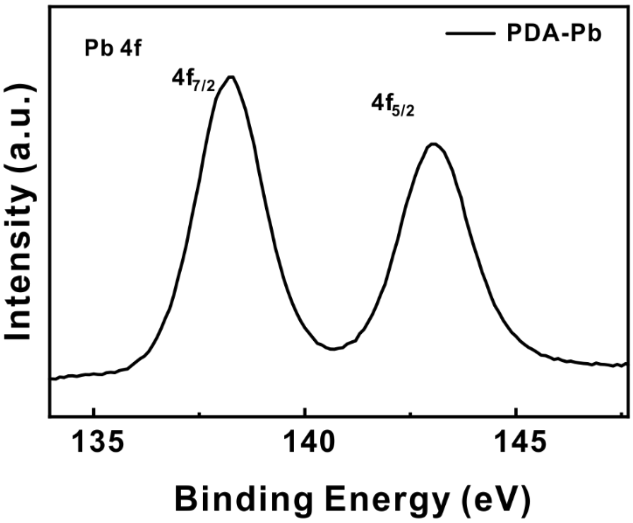

Figure S2. The high-resolution XPS Pb 4f spectra of the PDA-Pb.

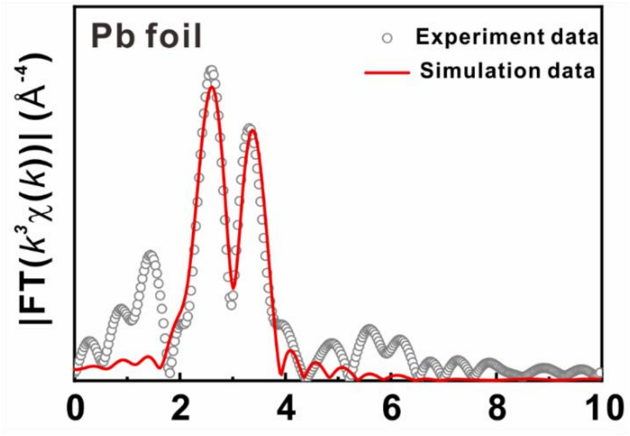

Figure S2. The Fourier transform (FT) EXAFS spectra and the corresponding fitting curve of Pb foil.

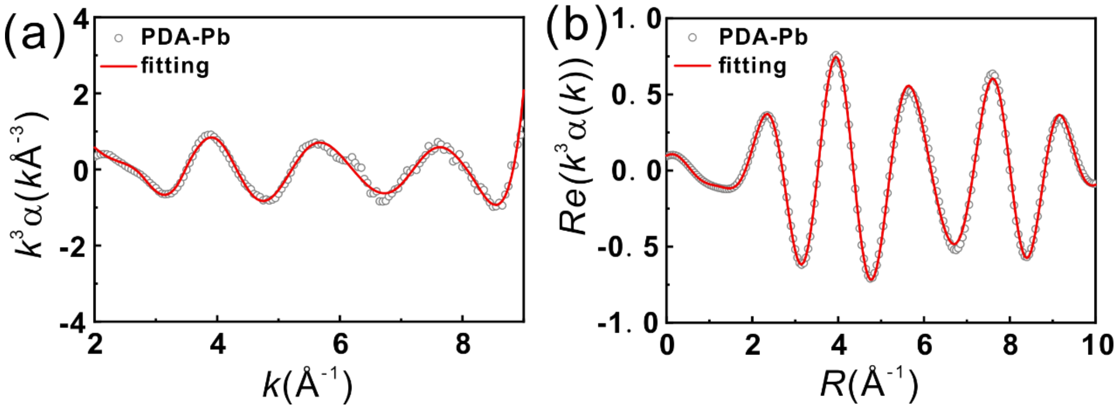

Figure S4. (a) The $k^3\chi(k)$ oscillations and corresponding fitting (b) The $Re(k^3\chi(k))$ oscillation curves and corresponding fitting of Pb L_3-edge EXAFS analysis for PDA-Pb.

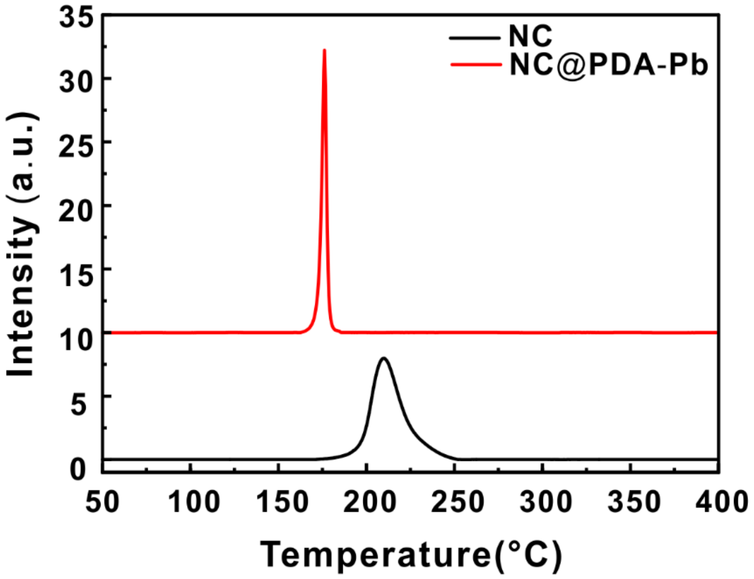

Figure S5. The DSC profiles of the thermal decomposition of nitrocellulose.

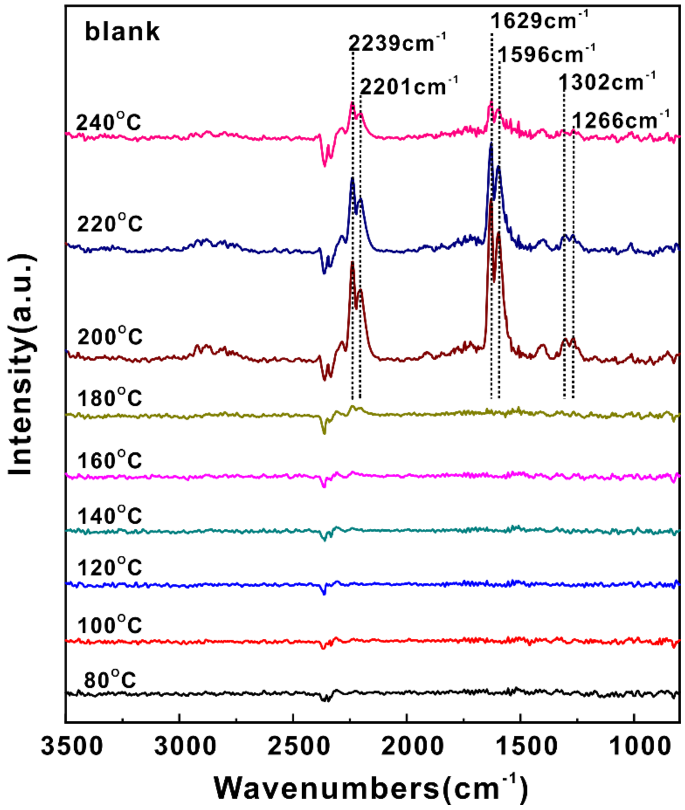

Figure S6. FTIR spectra of the gaseous decomposition products of RDX.

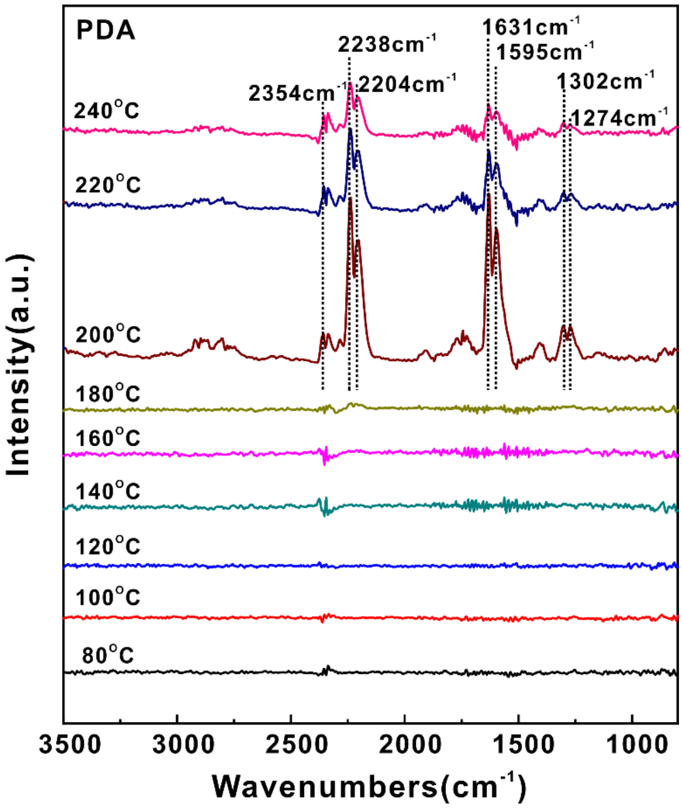

Figure S7. FTIR spectra of the gaseous decomposition products of RDX@PDA.

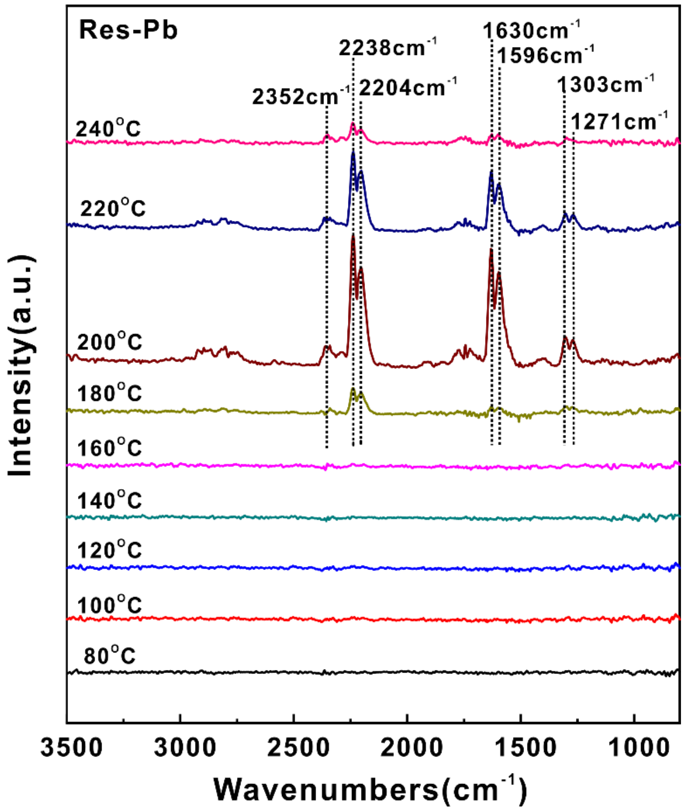

Figure S8. FTIR spectra of the gaseous decomposition products of RDX/Res-Pb.

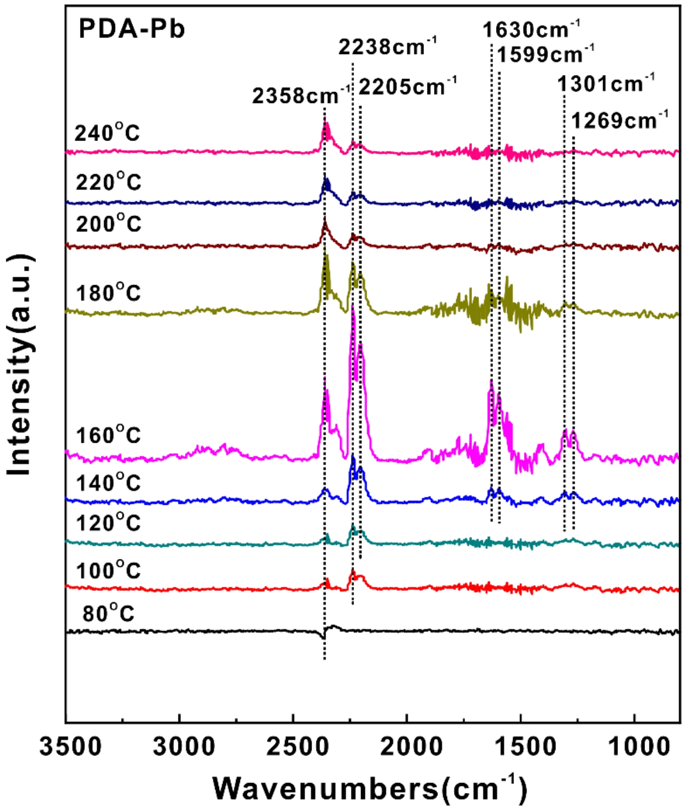

Figure S9. FTIR spectra of the gaseous decomposition products of PDA-Pb@RDX.

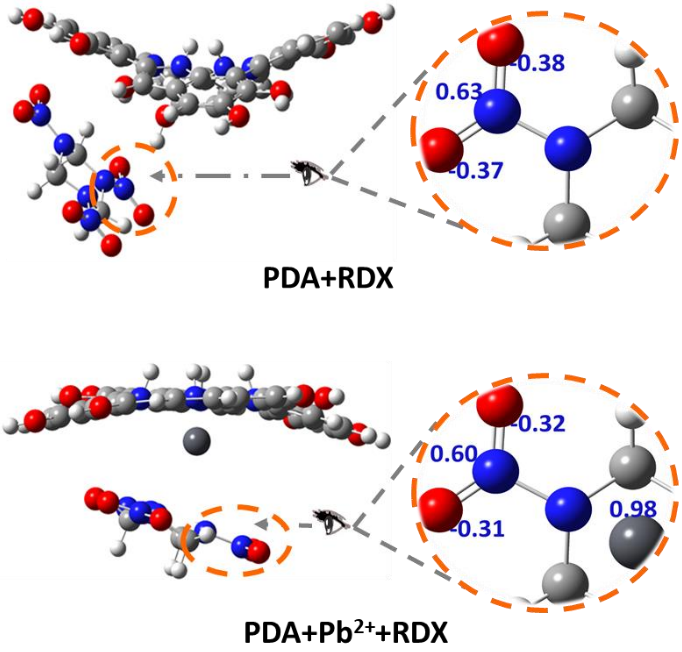

Figure S10. The charge distribution of the activated part of PDA+RDX and PDA+Pb^{2+}+RDX.

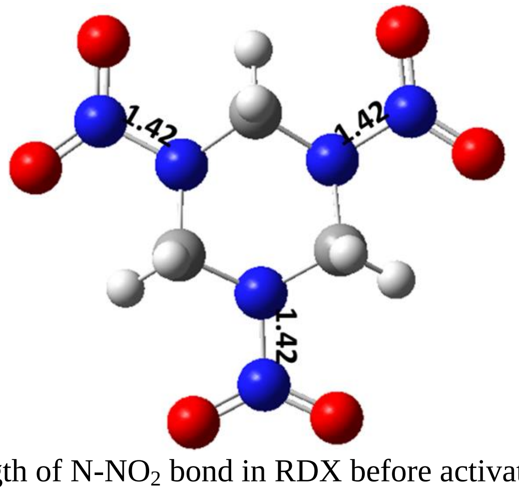

Figure S11. The bond length of N-NO_2 bond in RDX before activation

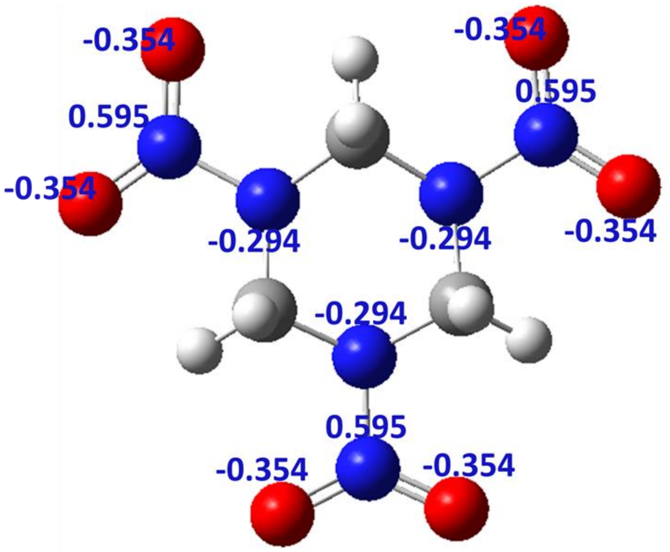

Figure S12. The charge distribution of N and O in RDX before activation

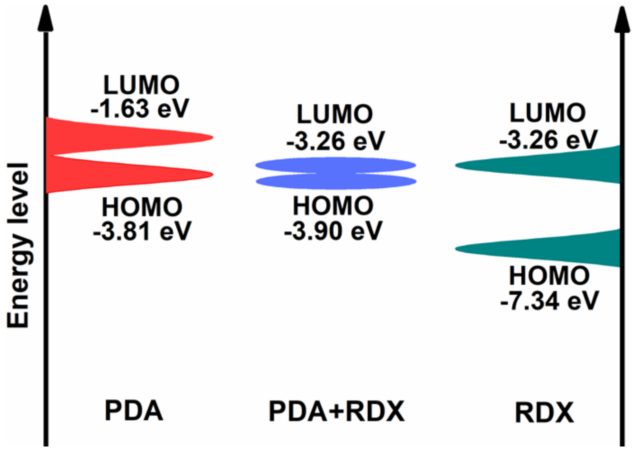

Figure S13. The relative position of the LUMO and HOMO of PDA with RDX before and after binding.

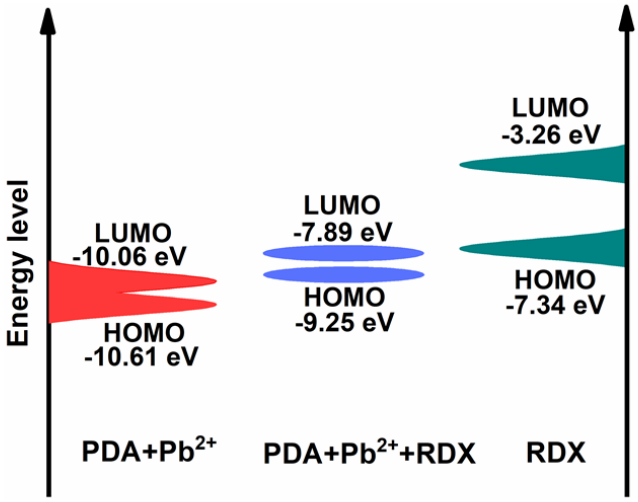

Figure S14. The relative position of the LUMO and HOMO of PDA+Pb^{2+} with RDX before and after the binding

Table S1. EXAFS fitting parameters for the Pb L_3-edge spectra (S_0^2=0.893).

Sample	Shell	N^a	R(Å)^b	σ^2(Å^2)^c	ΔE_0(eV)^d	R factor
Pb foil	Pb-Pb	12	3.39	0.025	-7.4	0.0068
PDA-Pb	Pb-N	2	2.46	0.0038	10.3	0.0051
	Pb- N	2	2.70	0.0079		

$^a N$: coordination numbers; $^b R$: bond distance; $^c\sigma^2$: Debye-Waller factors; $^d\Delta E_0$: the inner potential correction. R factor: goodness of fit. S_0^2 was set to 0.893, according to the experimental EXAFS fit of Pb foil reference by fixing the coordination number as the known crystallographic value.

Table S2. The detailed burning rates at various pressures.

Samples	Burning rate r (mm/s) at different pressure (MPa)									
	2	4	6	8	10	12	14	16	18	20

blank	3.09	5.34	7.42	9.85	11.88	14.04	15.75	17.54	19.23	20.92
PDA	3.25	5.64	7.62	9.51	11.29	13.72	15.41	17.19	19.02	20.76
Res-Pb	5.65	6.72	8.18	9.81	11.66	13.83	15.9	18.07	20.39	22.57
PDA-Pb	14.98	16.79	18.43	19.82	21.03	21.93	22.42	22.71	22.77	23.15

Table S3. The detailed pressure exponents at various pressures.

Samples	Pressure exponent *n* at different pressure (MPa)									
	2	4	6	8	10	12	14	16	18	20
blank		0.79	0.81	0.98	0.84	0.92	0.74	0.81	0.78	0.8
PDA		0.79	0.74	0.77	0.77	0.65	0.81	0.87	0.91	0.88
Res-Pb		0.25	0.48	0.63	0.77	0.94	0.9	0.96	1.02	0.94
PDA-Pb		0.16	0.23	0.25	0.26	0.23	0.14	0.09	0.02	0.16
